# Letter from New York

**Published:** 1875-08

**Authors:** 


### LETTER FROM NEW YORK.

NEW YORK, JUNE 1, 1875.

New York is fast becoming the center of medical instruction of this continent. Philadelphia, which, since the foundation of our government, has had the distinction of occupying that position, must now look to her honors, and increase her energy if she wishes to save them.

It is true, at this time, the colleges are closed, and there is not much activity anywhere in the matter of medical teaching, but even in the dull season what is only repose in New York more resembles lethargy in the Quaker City.

The great advantage of New York over other places to the student of medicine, especially the advanced student, is the opportunities he has of pursuing any special line of study he may select. In this particular it resembles the great European cities, especially the German, more than any other city in America; and, in fact, most of the teachers are imported from Germany, though many native Americans are not wanting who are reckoned high as teachers. It is needless to mention all the special institutions where thorough instruction can be obtained, as the names are familiar to most of your readers. There are two private institutions, however, which are doing a good and thorough work, whose merits need only be known to be appreciated. One of them, Prof. H. Knapp's Ophthalmic and Aural Institute, is, I presume, not unknown to many readers of the RECORD, as the Professor's fame has spread very rapidly during the six years of his residence in America. It is, I believe, with one exception, the only private ophthalmic hospital in America, and in point of excellence it will compare favorably with any in the world. The dispensary furnishes a large amount of material for clinical instruction in diseases of the eye and ear, and this is used to the best advantage by Dr. Knapp and his assistants. Nowhere in America, certainly, can ophthalmology and otology be more thoroughly studied than at this institution. We may add, too, that the "archives of ophthalmology and otology," of which Dr. Knapp is the chief editor, is doing much to give our country a position in science abroad, and furnishes a convenient opportunity to American ophthalmologists to place the results of their work upon record, and thus indirectly proves a stimulus to labor in the scientific field.

The other institution is less widely known, but that is simply because it is not as old. Its name is the New York Histological Institute, and its age is now about eight months. It is under the charge of Dr. C. Heitzmann, for many years assistant to Prof. Hebra, the great Vienna dermotologist, and a co-worker with Stricker, the renowned histologist of the same city, whose work has become an authority on the subject of which it treats.

This institute is conducted on the same principle as Stricker's, and the Brown Institution in London, but is more extensive, embracing pathological as well as physiological histology. There is every facility for the study of histology in all its branches, and the Doctor is not only willing but eager to communicate all the knowledge he is possessed of. And he is one of the most genial men in the world. To work in his laboratory, is a feast indeed. He is preparing for publication a work on histology, which he hopes to have out by autumn. It will be different from anything at present before the profession, as it will comprise only personal and original investigations, and will not be a resume of other men's work. I saw in a recent number of the Boston *Medical and Surgical Journal* that the instruction in Stricker's institute was not all that it might be, and was somewhat unsatisfactory to Americans who went to Vienna to study histology. However this may be, it is certain that Amercans hereafter need go no further than New York to have as efficient instruction in this line as can be procured at any other one place in the world. This new institute of Dr. Heitzmann's is welcomed by the profession of New York as quite an addition to scientific medicine in the metropolis.

The Doctor, in addition to his eminence as a dermatologist and histologist, is also a most accomplished draughtsman, and many of the fine chromolethographs, illustrative of the work done by various eminent men in Vienna, in ophthalmology, otology, dermatology, are the products of his pencil. He showed me his fine chromolethograph of the famous tattooed man. Some, at least, of your readers have heard of the two Greeks who went into Asia with intentions on the rich mines of gold there, who were taken by the Asiatics and disposed of in a remarkable manner, at least one of them was. One was supposed to be instantly killed, while the other had his entire body covered with representations of all kinds of beasts, birds and reptiles. Not the smallest portion of his body but what is covered with the tattooing. A most peculiar looking picture it is truly.

There are other matters I would liketo speak of, as, for instance, the opening of the new Academy of Medicine, but I have already extended this letter beyond reasonable limits. S. M. B.

